# Rigid External Distraction with Intranasal Bone-borne Traction Hooks for Midfacial Hypoplasia

**DOI:** 10.1038/s41598-018-28068-8

**Published:** 2018-07-02

**Authors:** Yilue Zheng, Haizhou Tong, Ningbei Yin, Feng Niu, Zhenmin Zhao, Tao Song

**Affiliations:** 1 Center for Cleft Lip and Palate Treatment, Plastic Surgery Hospital, Chinese Academy of Medical Sciences and Peking Union Medical College, Beijing, China; 2 Department of Stomatology, Beijing Children’s Hospital, Capital Medical University, Beijing, China

## Abstract

Rigid external distraction is currently performed to correct cases of severe maxillary hypoplasia. As an improvement of this technique, we propose the use of an intranasal bone-borne traction hook. This study is a retrospective chart review of the intranasal bone-borne traction hooks used in the treatment of severe maxillary hypoplasia. There were 110 patients treated with the hooks from 2005 to 2017. The maximum traction force was 7.75 kg, and there were few complications encountered during distraction. There were 76 patients who had the hooks removed under local anaesthesia. A cephalometric analysis was conducted in 56 patients. The average advancement of A-point was 9.9 ± 4.2 mm, 8.4 ± 2.5 mm, 11.0 ± 3.7 mm, 17.9 ± 4.4 mm for the trans-sutural distraction osteogenesis (DO), maxillary anterior segment DO, Le Fort I osteotomy DO and Le Fort III/II osteotomy DO, respectively. The average changes of sella-nasion-point A (SNA) were 8.89 ± 4.30 degrees, 8.21 ± 3.17 degrees, 10.49 ± 3.26 degrees, and 15.10 ± 4.00 degrees, respectively. The A point-nasion-B point (ANB) also showed increases in all procedures with P < 0.001. In conclusion, this technique sufficiently advances the midface and ensures the scars are concealed in the nasal base. The traction hook can bear a large traction force, causes minimal complications and is easily removed.

## Introduction

Midfacial hypoplasia is a common dentofacial malformation associated with several congenital diseases, such as cleft lip and palate and Crouzon syndrome; this condition usually leads to significant problems. Patients often present with class III malocclusion, severe concave facial appearance, and/or teeth crowding^1^. Patients may have difficulty breathing, and the majority of cases require orthognathic surgery to achieve aesthetic and functional results.

In 1997 Polley and Figueroa^2^ first introduced the use of a rigid external distraction (RED) device to treat severe maxillary hypoplasia using distraction osteogenesis (DO). This approach was shown to be an effective technique. RED involves an external bow fixed to the cranium by screws and an intraoral splint cemented to the maxillary first molars. External traction hooks with eyelets are soldered to the splint via surgical wires. Due to the dental compensation and deformation of traction hooks and intraoral splint, several authors have suggested directly attaching the halo device to the bone with fixation plates^3,4^. However, increased traction forces may cause the skeletal plates to become loose due to bone resorption around fixation screws^5^, and there is a high risk for loss of osseointegration when tipping forces exceed 600 cN mm^6^. Therefore, we propose an intranasal bone-borne traction hook to replace the fixation plate. This technique sufficiently advances the midface while ensuring that scars are concealed in the nasal base; this technique also eliminates the need for an intraoral splint.

## Results

### Clinical review

There were 110 patients with midfacial hypoplasia treated using intranasal bone-borne traction hook distraction from 2005 to 2017. There were 73 patients who underwent trans-sutural DO, 19 patients who underwent maxillary anterior segment DO, 11 patients who underwent Le FortIDO, 6 patients who underwent Le Fort IIIDO and 1 patient who underwent Le Fort IIDO.

Within the cohort of 110 patients, 46 underwent trans-sutural DO and had a detailed record of traction force evaluated every 1 to 3 days. The average unilateral maximum traction force was 4.75 ± 1.03 kg (range, 3 to 7.75 kg)(Table 1).

**Table 1:** Patient characteristics.

Operation			Trans-Sutural DO	MASDO	Le Fort IDO	Le FortIII DO	Le FortIIDO
Objective							
Number			73	19	11	6	1
Age (Mean, range, y)			11.2 7–16	19.7 18–22	18.1 13–23	14.0 9–20	16.0 /
Cause			CLP	CLP/MH	CLP	Crouzon syndrome	Binder syndrome
Complication (No.)			Loosening of the cranial frame(4) Maxillary fracture(3) Infection(1)	None	None	None	None
Traction force (Mean ± SD, kg) (No.)			4.75 ± 1.03(46)	/	/	/	/
Advancement (Mean, mm) (No.)			10.4(28)	8.4(12)	11(9)	17.3(6)	21.5(1)
Removing the hooks	LA	Without incisions	41	15	1	2	None
		With incisions	13	4	None	None	None
	GA	With other procedures	12	None	10	3	1
		Without other procedures	7	None	None	1	None

**CLP**, cleft lip and/or palate; **DO**, distraction osteogenesis; **MH**, maxillary hypoplasia; **LA**, local anaesthesia; **GA**, general anaesthesia.

There were 76 patients who had the hooks removed under local anaesthesia. There were another 34 patients requiring general anaesthesia. Within 34 patients, there were 12 patients who received treatment for secondary deformities of cleft lip, 11 patients who treated for bilateral sagittal split ramus osteotomy, 1 patient who received the maxilla internal fixation and 1 patient who received repair of palatal fistula (Table 1).

### Imaging Analysis

Amongst the 110 patients, there were 56 patients who underwent analysis of lateral teleradiographs. In some patients, the final maxilla position was decided by distraction length measured though the changes of spring length and occlusion relation. There were also patients who received cephalometric X-rays with external distraction devices, resulting in absence of a scale ruler. There were also several patients who had computed tomography without X-rays.

In this study, 28 of 73 patients received cephalometric analysis during the treatment of trans-sutural DO. The skeletal angular and linear changes are shown in Table 2. The SNA was significantly increased when pre-operative images were compared with those obtained immediately post-operatively, and the average increase was 9.11 ± 4.33 degrees (p < 0.001). The ANB also substantially increased; the average increase was 9.17 ± 4.29 degrees (p < 0.001). The mean horizontal advancement of A-point was 9.9 ± 4.2 mm (range, 2.6 to 20.8 mm).

**Table 2:** Changes in the facial skeleton after trans-sutural distraction osteogenesis.

Patients	Age (y)	Sex	Diagnosis	Change in A point. Distance, mm	Change in SNA Degree	Chang in ANB degree
1	9	F	UCLP	17.3	12.83	12.41
2	17	M	UCLP	11.2	7.77	6.83
3	14	F	UCLP	8.2	5.58	8.83
4	8	F	UCLP	13.6	10.00	11.91
5	13	F	UCLP	12.4	12.71	9.15
6	13	M	UCLP	9.0	7.94	5.90
7	13	M	UCLP	12.5	12.58	11.83
8	13	M	UCLP	9.8	11.51	9.65
9	8	M	UCLP	4.1	3.11	5.59
10	9	M	UCLP	20.8	21.84	21.30
11	8	M	UCLP	19.0	12.59	11.28
12	12	M	UCLP	8.5	12.16	14.45
13	8	M	BCLP	9.7	5.18	2.19
14	7	M	BCLP	10.9	8.28	11.77
15	12	F	UCLP	13.4	13.24	14.79
16	11	F	UCLP	9.1	8.54	8.00
17	12	M	BCLP	2.6	1.14	2.26
18	8	M	BCLP	2.9	3.77	3.61
19	8	M	BCLP	7.5	7.36	4.90
20	9	M	BCLP	7.0	8.69	6.83
21	8	M	UCLP	10.4	12.34	13.40
22	7	M	UCLP	10.9	13.33	12.72
23	12	M	UCLP	6.7	5.21	7.44
24	11	F	BCLP	9.9	12.30	11.33
25	11	F	BCLP	9.5	10.17	12.03
26	10	F	UCLP	4.5	2.77	3.97
27	9	F	BCLP	7.1	7.13	6.22
28	10	F	UCLP	8.4	5.05	6.12
Mean ± SD	10.4 ± 2.4	/	/	9.9 ± 4.2	9.11 ± 4.33	9.17 ± 4.29
*P* value	/	/	/	<0.001	<0.001	<0.001

**F**, female; **M**, male; **UCLP**, unilateral cleft lip and palate; **BCLP**, bilateral cleft lip and palate.

***P*** value, Paired *t*-test of pre- and post-operation with a 5% level of significance.

We conducted cephalometric analysis in twelve patients during the treatment of maxillary anterior segment DO (Table 3). The SNA was increased by an average of 8.20 ± 3.17 degrees (range, 3.01 to 13.80°). The ANB was increased by an average of 9.84 ± 3.65 degrees (range, 6.01 to 18.83°). The mean horizontal movement of A-point was 8.4 ± 2.5 mm (range, 4.1 to 11.4 mm).

**Table 3:** Changes in the facial skeleton after maxillary anterior segment distraction osteogenesis.

Patients	Age (y)	Sex	Diagnosis	Change in A point. Distance, mm	Change in SNA Degree	Chang in ANB degree
1	20	M	UCLP	4.1	3.01	6.01
2	17	M	UCLP	8.2	7.45	7.49
3	24	M	UCLP	11.2	12.97	13.10
4	23	F	UCLP	5.4	4.76	8.67
5	25	F	MH	5.0	7.80	12.29
6	18	M	MH	5.7	6.97	8.14
7	22	M	UCLP	10.2	13.80	13.58
8	22	F	UCLP	9.8	7.17	7.10
9	22	F	UCLP	9.7	12.78	18.83
10	20	F	UCLP	8.8	7.85	8.52
11	19	M	UCLP	11.4	6.69	6.99
12	18	F	BCLP	10.9	7.17	7.33
Mean ± SD	20.8 ± 2.4	/	/	8.4 ± 2.5	8.20 ± 3.17	9.84 ± 3.65
*P* value	/	/	/	<0.001	<0.001	<0.001

**F**, female; **M**, male; **UCLP**, unilateral cleft lip and palate; **BCLP**, bilateral cleft lip and palate; **MH**, maxillary hypoplasia; ***P*** value, Paired *t*-test of pre- and post-operation with a 5% level of significance.

Eleven patients accepted Le Fort Iosteotomy DO. The skeletal angular and linear changes were measured in nine patients (Table 4). The SNA was improved with an average change of 10.48 ± 3.27 degrees (p < 0.001). The ANB was also improved, with an average change of 15.84 ± 4.67 degrees (p < 0.001). The mean horizontal advancement of A-point was 11.0 ± 3.7 mm (range, 6.0 to 17.8 mm).

**Table 4:** Changes in the facial skeleton after Le Fort I osteotomy distraction osteogenesis.

Patients	Age (y)	Sex	Diagnosis.	Change in A point. Distance, mm	Change in SNA Degree	Chang in ANB degree
1	22	F	UCLP	6.0	5.78	5.67
2	19	F	UCLP	9.1	9.47	19.74
3	18	M	UCLP	14.0	12.99	13.53
4	19	M	BCLP	17.8	11.23	23.44
5	16	M	BCLP	14.2	11.66	15.97
6	23	M	BCLP	9.6	9.28	13.22
7	18	M	BCLP	8.5	9.55	15.55
8	17	F	UCLP	12.7	17.49	18.24
9	18	M	UCLP	7.2	6.85	17.19
Mean ± SD	18.9 ± 2.1	/	/	11.0 ± 3.7	10.48 ± 3.27	15.84 ± 4.67
*P* value	/	/	/	<0.001	<0.001	<0.001

**F**, female; **M**, male; **UCLP**, unilateral cleft lip and palate; **BCLP**, bilateral cleft lip and palate.

***P*** value, Paired *t*-test of pre- and post-operation with a 5% level of significance.

**Figure 1:**
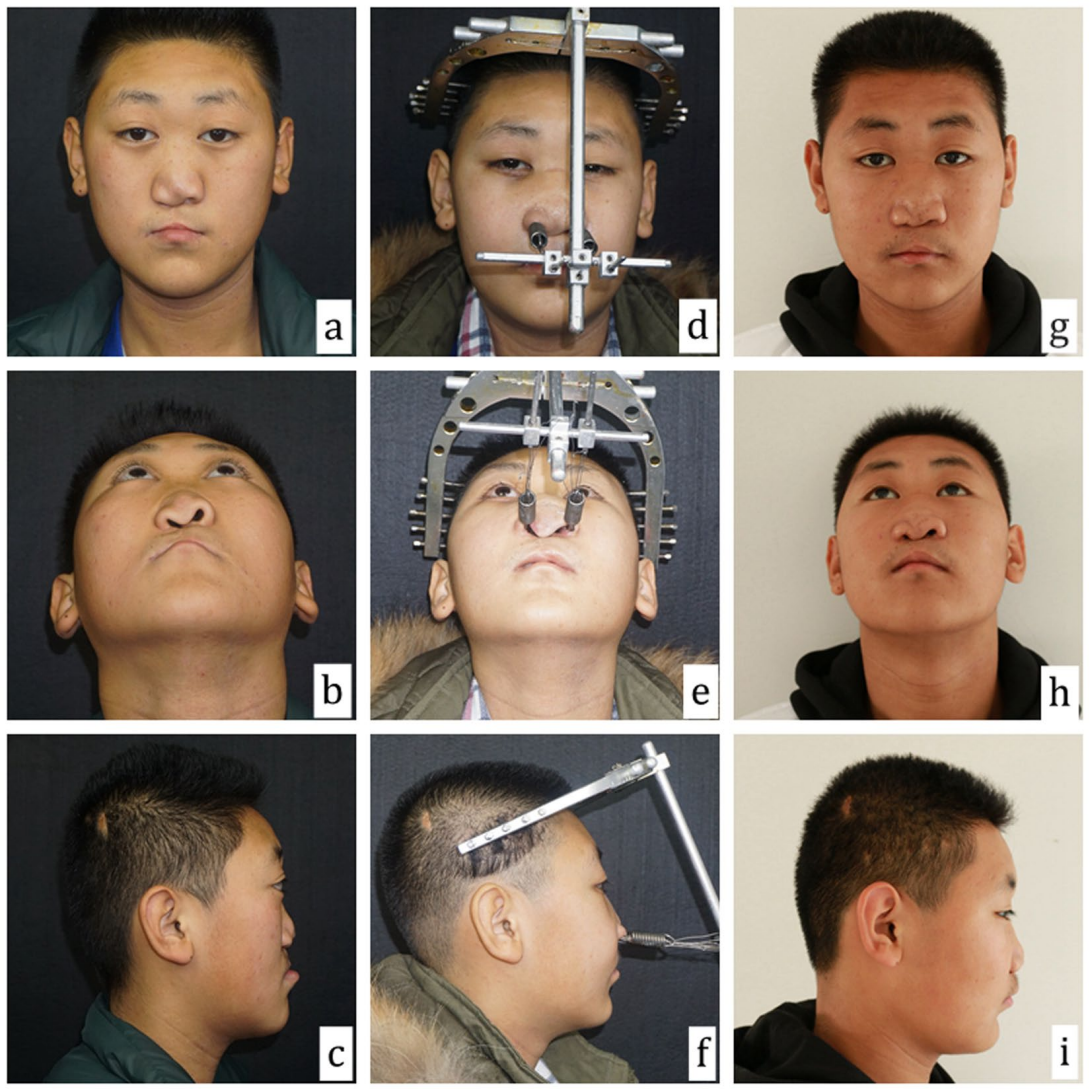
Binder syndrome patient with a right-side unilateral cleft and midfacial hypoplasia. Le Fort II distraction osteogenesis was performed to draw the midface out. The preoperative photographs show severe depression of the midface (**a**–**c**). After 6 months of distraction osteogenesis the patient achieved a harmonious face (**d**–**f**). The lateral cephalometric analysis showed that the maxilla was advanced 21.5 mm and the SNA angle changed from 71.79 degrees to 90.87 degrees. After 12 months, a good appearance was still maintained (**g**–**i**).

There were 6 patients who underwent Le Fort III osteotomy DO and one patient who underwent Le Fort II osteotomy DO (Fig. 1). The outcome of cephalometric analysis is shown in Table 5. The SNA had an average improvement of 15.10 ± 4.00 degrees (p < 0.001). The ANB had an average improvement of 16.58 ± 2.30 degrees (p < 0.001). The mean horizontal advancement of A-point was 17.9 ± 4.4 mm (range, 11.1 to 25.1 mm).

**Table 5:** Changes in the facial skeleton after Le Fort III/II osteotomy distraction osteogenesis.

Patients	Age (y)	Sex	Diagnosis	Change in A point. Distance, mm	Change in SNA Degree	Chang in ANB degree
1	10	M	Crouzon syndrome	18.3	19.86	21.46
2	16	M	Crouzon syndrome	25.1	17.56	15.64
3	15	F	Crouzon syndrome	11.1	12.56	14.59
4	18	F	Crouzon syndrome	13.1	7.40	14.68
5	20	F	Crouzon syndrome	18.9	13.89	15.01
6	9	M	Crouzon syndrome	17.3	15.34	16.64
7	13	M	Binder syndrome	21.5	19.08	18.06
Mean ± SD	14.4 ± 3.7	/	/	17.9 ± 4.4	15.10 ± 4.00	16.58 ± 2.30
*P* value	/	/	/	<0.001	<0.001	<0.001

**F**, female; **M**, male; ***P*** value, Paired *t*-test of pre- and post-operation with a 5% level of significance.

### Complications

There were four patients who had complications of loose cranial frame, and there were three patients with traction hook failure due to pyriform rim fracture during the treatment of trans-sutural DO. One patient developed an infection, which resolved after the hooks were removed. These complications occurred during the early treatment period when this technique was still under development. There were no complications in the other treatments.

## Discussion

External DO is a well-established treatment for midfacial hypoplasia^7,8^. In our study, the cephalometric analysis showed increases in horizontal advancement, SNA and SNB. The treatment sufficiently advances the midface. DO gradually advances the bony structures of the midface, which allows for progressive osteogenesis and adaptation of the surrounding soft tissues. The gradual surgical movement requires lower counterforce to pull the maxilla into the appropriate position, which makes the maxilla more stable than in traditional Le Fort Iosteotomy^9,10^.

External distraction has generally involved using traction hooks with eyelets and an intraoral splint to attach the maxilla though the dentition. The intraoral wires or splint can be unwieldy and uncomfortable for patients and impede oral hygiene. Additionally, in patients with unsound dentition and severe cleft who are treated with the use of dental anchorage with intraoral orthodontic splint, it is difficult to secure sufficient anchorage. An alternative option involves transcutaneous attachment of the distractor to the maxilla. However, this can cause conspicuous scars^11^. The study by Yu and Woo^12^ minimized the visibility of pin site scars in the alar crease, but scars were still distinguished on the face, especially when infections occurred at the skin site. The intranasal bone-borne traction hook concealed the scars in the nostril base and had little influence on appearance. In contrast to the intraoral splint approach, the new technique can be used for edentulous patients and for the treatment of cleft lip and palates.

Soft tissue scars such as palatal scars, cheeks, and upper lip scars resist maxilla advancement. The majority of patients requiring DO are likely to have significant resistance, especially due to scarring from cleft palate repair. Thus, the expected traction force would be substantial. Sawada *et al*. reported the traction force in Le Fort I DO ranged from 13.4 to 26.8 N^13^. In our trans-sutural DO, the traction force was much larger, with an average of 4.75 ± 1.03 kg. Severe bowing of the external traction hooks could happen, which may result in inappropriate rotation of the osteotomized bony segment and an unwanted dentoalveolar effect^4,13^. Furthermore, unexpected loosening of fixation plates could occur. Schulten *et al*.^5^ reported bone resorption around fixation screws supporting distraction devices in animals, and the resorption increased with time. Buchter *et al*.^6^ investigated the load-related bone modelling at the interface of orthodontic micro-implants and found there was a high risk of the loss of osseointegration when tipping forces exceeded 600 cN mm. The intranasal bone-borne traction hooks with canine pillars as anchorage could bear a large traction force, as was previously demonstrated for cases involving trans-sutural DO^14^. Although the hooks were easily removed without any incisions under local anaesthesia, they rarely fell out during distraction with the continuous traction force.

The traction hooks failed in only three patients, and the failures were due to rapid distraction-induced pyriform rim fracture. These fractures happened during the early study period when this technique was still under development. The current strategy uses a variable distraction rate of 1 to 2 mm every 1 to 3 days, and there have been no fractures. Infection is a potential complication when using any external distraction device, including intranasal bone-borne traction hooks. Only one patient developed an infection, which resolved after the hooks were removed. Several patients complained of pain during distraction. This complication was easy to resolve by decreasing the traction force and rate. Several studies have been published on the topic^14–17^, and all have demonstrated the usefulness of this approach.

In conclusion, the use of intranasal bone-borne traction hooks offers advantages over the use of an intraoral splint and fixation plates. The hooks produce scars that are better concealed, bear a larger distraction force and are easily removed. This approach caused minimal complications and is a reasonable option for midface DO.

## Methods

This retrospective study was designed to investigate the effectiveness of intranasal bone-borne traction hooks in DO. We reviewed all the patients with intranasal bone-borne traction hooks for distraction osteogenesis in the Center of Cleft Lip and Plate, Plastic Surgery Hospital, Chinese Academy of Medical Sciences and Peking Union Medical College from 2005 to 2017.

### Ethics

The Plastic Surgery Hospital, Chinese Academy of Medical Sciences, and Peking Union Medical College ethics committee approved the study, which was performed according to the principles of the Declaration of Helsinki. Written informed consent was obtained from the patients or from the guardians of the patients younger than 18 years. All identifying images released in this article were authorized for publication by the patient and his guardians.

### Surgical technique

**Figure 2:**
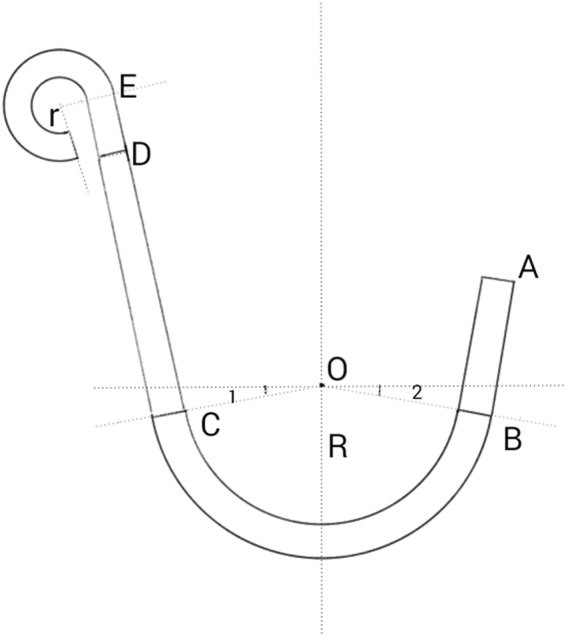
Detail parameters of the traction hook. AB = 6 mm, CD = 11 mm, DE = 2.75 mm, R = 6 mm, r = 1.25 mm, ∠1 = ∠2 = 10°, Φ = 1.5 mm

The distraction system consisted of an RED (Cibei Medical Treatment Appliance Co., Ltd., Ningbo, China), and nickel–titanium “shape memory alloy” spring and nickel–titanium bone-borne traction hooks (diameter: 1.5 mm; GEE Co., Beijing, China)^12–14^. The length of the traction hooks could be adjusted to suit different patients. The spring generated a continuous force of approximately 250 g/mm. The detailed parameters are shown in Fig. 2.

**Figure 3:**
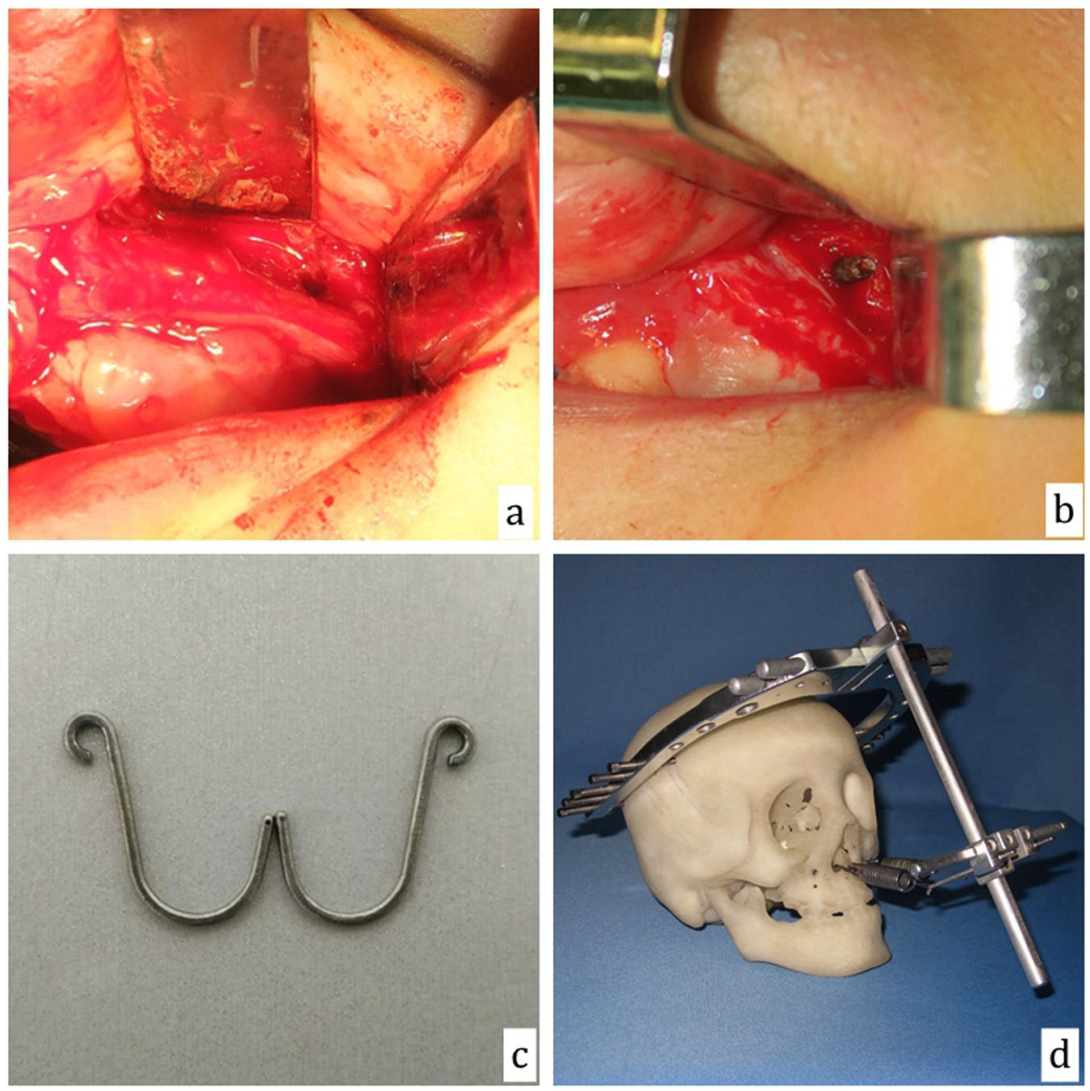
A hole was drilled through the lateral nasal wall (**a**) and the traction hook was introduced through the hole (**b**). Intranasal bone-borne traction hooks (**c**) and connections between rigid external distraction device components (**d**).

Maxillary vestibular incisions were made, and the buccal tissue was moved to expose the pyriform rim and canine root. A thick fissure burr was used to drill a hole approximately 1 cm outside the lateral pyriform rim and 5 mm above the teeth apices through the lateral nasal wall. The traction hooks were introduced through the hole and the caudal ends extended out from the nostril base. The RED cranial frame was applied at 20–30° upward from the Frankfurt horizontal plane with the vertical rod 10–12 cm anterior of the nostril base. The spring was then usually used to connect the hooks to the RED device, especially in the treatment of trans-sutural DO. In several maxilla osteotomy DO cases, we also used firm surgical wires to directly connect the traction hooks and RED device (Fig. 3).

### Distraction Protocol

The distraction commenced immediately after the operation with an initial force of 750 g on each side for the treatment of trans-sutural DO. An adaptive period of 3–5 days followed, and then the traction force was increased gradually with a variable distraction rate of 1 to 2 mm every 1 to 3 days. The traction force was recorded in some patients. When there was significant advancement of the maxilla observed the force was increased slowly to the maximum and was maintained until the required advancement was achieved. Then, the distraction was followed by a consolidation period of 1–3 months with gradually decreased traction force.

In the treatment of Le Fort I/II/IIIand maxillary anterior segment DO the distraction was initiated with a variable rate of 1 to 2 mm every day after a 5–7-day latency period. The end of the distraction was determined by clinical judgement and cephalometry.

After a consolidation period the RED frame and traction hooks were usually removed in the surgery room under local anaesthesia. We directly removed the hooks along their curved shape through the nostril base without maxillary vestibular incisions. If the operation time was over five minutes, it indicated difficulty removing the traction hooks and maxillary vestibular incisions were made to complete the removal. Several patients chose to remove the traction hooks under general anaesthesia when they underwent treatment of secondary deformities six months or twelve months later. Several patients also chose general anaesthesia because of their young age and limited cooperation.

### Imaging Analysis

A single examiner performed all blind assessments based on a manual cephalometric analysis of lateral teleradiographs. The radiographs were obtained both pre- and post-operatively immediately after DO. The bony landmarks and reference lines used for analysis included the following points: sella (S), nasion (N), subspinale A-point (A), supramental B-point (B). The horizontal (X) reference line was constructed at 7 degrees relative to the sella-nasion (SN) line, and the vertical (Y) reference line was through the sella perpendicular to the horizontal reference. The distance from Y line to point A was measured both pre- and post-operatively. All the lateral cephalographs were traced and superimposed using the cranial base points. The following angular measurements were also assessed: sella-nasion-point A (SNA), A point-nasion-B point (ANB). Skeletal angular and linear changes were recorded.

Data sharing statement: The data can be assessed: https://pan.baidu.com/s/1pLp7Sk7; Extra data are available by emailing songtao2059@163.com.

### Statistical analysis

The statistical analysis was performed using SPSS 20.0 (IBM Corp., Armonk, NY, USA). The same process was repeated 1 week later to assess measurement accuracy. The reliability analysis tests were used to assess the two sets of tracing measurements. Reliability was confirmed using a paired *t*-test with a 5% level of significance. The result showed no significant difference between the two sets of tracings. The paired *t*-test was used to analyse the difference of distance from Y line to point A, SNA and SNB between pre- and post-operation, with a 5% level of significance. The average advancements and changes of SNA and SNB for the four different procedures were described.
